# Health may be compromised by social interactions depending on culture among postpartum Arab and Jewish Israeli women

**DOI:** 10.1186/s12884-020-03168-4

**Published:** 2020-08-21

**Authors:** Sadie Puddister, Ola Ali-Saleh, Michal Cohen-Dar, Orna Baron-Epel

**Affiliations:** 1 School of Public Health, Faculty of Social Welfare and Health Sciences, University of Haifa, 31905 Mount Carmel, Haifa Israel; 2 Northern Region Health Office, Ministry of Health, Nazareth, Israel

**Keywords:** Postpartum period, Social interaction, Maternal health, Culture, Customs

## Abstract

**Background:**

Social support is generally perceived to facilitate health in postpartum women; however, previous research shows that this is not always true. Social interactions intended to provide support can be perceived as negative and in turn, may have negative impacts on maternal health. The purpose of the present study was to asses if social support and negative interactions at one month after childbirth can predict maternal health four months after childbirth, and if this relationship is influenced by culture.

**Methods:**

This prospective longitudinal cohort study included randomly selected Arab (*n* = 203) and Jewish (*n* = 202) women who attended Mother and Child Health Clinics in Northern Israel one month after giving birth. The women were interviewed at one and four months after childbirth using a questionnaire including measures of health (self-reported health (SRH) and health problems), socioeconomic and demographic status, obstetric characteristics, social support, negative social interactions and perceptions of customs and traditions intended to help the mother cope after childbirth. Multivariable regressions were run to identify the variables predicting health four months after childbirth.

**Results:**

The response rate for both interviews was 90%. Negative social interactions one month after childbirth significantly predicted health problems in Arab and Jewish women (Beta 0.20 and 0.37 respectively) and SRH among Arab women only (odds ratio (OR) 0.32, confidence interval (CI) 0.19–0.54) four months after childbirth. Social support at one month after childbirth significantly predicted better SRH in both Jewish and Arab women four months after childbirth (OR 2.33, CI 1.38–3.93 and 1.59, CI 1.01–2.46 respectively) and fewer health problems only among Jewish women (Beta − 0.37).

**Conclusions:**

Social support and negative social interactions appear to be predictive of health in postpartum women. Associations varied between Arabs and Jews, indicating that social support may be more important for predicting health among Jewish women and negative interactions may be more important among Arab women. Healthcare practitioners should be aware of the cultural context and social circumstances of postpartum women to ensure they receive the social support and care they need.

## Background

The postpartum period, although a wonderful time for a family, is a vital transitional period that is often accompanied by significant mental and physical stress for new mothers [1, 2, 3]. In fact, the majority of women experience either or both mental and physical health problems in the first year after childbirth [4, 5].

In order to help mothers cope with the newborn and household chores, various cultures have customs intended to provide support. This social support is considered to be beneficial to the health of both mother and child [6, 7, 8, 9, 10]. However, this is not always the case. Lee and colleagues (2004), described postpartum social support as a double-edged sword, providing practical support on one hand and a source of interpersonal conflict on the other [11]. This conflict, along with feelings of obligation and stress, is associated with the exacerbation of health problems and can negatively impact both mother and child [12, 13, 14]. The present study was based on Brooks and Dunkel Schetter’s (2011) conceptual framework [15] that summarizes social negativity influencing health as conflict, insensitivity and interference.

Israel, is a culturally diverse country comprised largely of an individualistic Jewish society (74.4%) and a more collectivistic, yet transitioning, Arab society (20.9%) [12]. In collectivistic cultures, the family and social circles are valued above individual needs, whereas in individualistic cultures, individuals are autonomous units [16] with more freedom to act independently and focus on personal preferences [17]. Between the two cultures, customs, traditions and expectations of new mothers after childbirth can vary greatly, resulting in very different postpartum experiences. For example, in Arab culture, the postpartum period is perceived as a stage in which the woman is vulnerable and requires special care and assistance. Often, women practice confinement by staying home for 40 days after giving birth [18]. There, they are visited frequently by relatives, friends and community members who help with household tasks and infant care, and provide social recognition through rituals, gifts and the preparation of special foods [19, 20, 18]. These traditions do not play out in the Jewish community in Israel.

Despite vast cultural differences both within and between these two groups, they are serviced by the same public healthcare system [21]. To ensure all members of the population receive adequate care, it is important for healthcare providers to be aware of cultural differences that impact health outcomes.

Prior to this study, a qualitative investigation of the impact of cultural customs intended to provide support for Arab-Israeli women during the postpartum period on the women and their families was conducted. The study identified both negative and positive social interactions to be present and influential on the women’s mental and physical health during the postpartum period [22]. The present study is a quantitative follow up of this research.

The purpose of the present study was to investigate the relationship between social interactions and the mental and physical health of Arab and Jewish women, with the objective of identifying if social support, negative social interactions and attitudes towards social customs at 1 month after childbirth can predict health 4 months after childbirth. It was hypothesized that higher levels of social support and positive attitudes towards customs at 1 month postpartum would predict positive health outcomes at 4 months and social negativity and negative attitudes towards customs at 1 month postpartum would be predictive of poorer health outcomes at 4 months. As well, it was hypothesized that differences in these associations would exist between Arab and Jewish women due to societal and cultural differences.

## Methods

The present study was a prospective longitudinal cohort study of women from one to 4 months after childbirth. Arab and Jewish women residing in towns or villages in the northern part of Israel were recruited for interviews 1 month after childbirth at the Ministry of Health’s (MOH) Mother and Child Health Clinics (MCHC), which 95 % of new mothers visit. The interviews were performed from June 2015 to January 2016.

Sampling was performed in two stages. First, towns and villages, both Arab and Jewish, were chosen randomly from the two to four socioeconomic status (SES) ranking of towns (a 10-rung scale where 1 is the lowest and 10 the highest) calculated by the Israel Bureau of Statistics for the year 2013 [23]. Most Arab towns are within this range of SES, therefore in order to match the two groups, a random selection was taken from Jewish towns and villages of the same SES rank. We chose one large Arab town (Nazareth), one large Jewish town (Afula), four Arab villages and three Jewish villages. All MCHCs in the chosen towns and villages were sampled. The second level of sampling included women visiting the MCHCs with their one-month-old baby. Nurses randomly recruited women answering the following inclusion criteria: mother, baby and all other children were healthy, the baby was born 1 month before and was delivered after the 37th week of pregnancy, the mother was living with a spouse, and gave informed consent to both the initial and follow-up interviews. Using a predetermined script, a research assistant explained the study and asked the women to give written informed consent. The women interviewed agreed to answer a second survey 3 months later. Those who agreed to answer the questionnaire were given it to fill out by a research assistant while waiting for their appointment with the nurse. Three months later they were contacted by phone and interviewed. The women had provided their telephone numbers for the follow-up interview on a separate form to maintain participant anonymity and a four-number identification code was added to the questionnaire to enable the coupling of the two interviews. The first interview lasted about 15–20 min and the second 8–10 min.

Research assistants were trained by one of the lead researchers who is proficient in both qualitative and quantitative research methods. As the first interview was self-administered, the assistant only answered questions if something was not understood. For the phone interview, research assistants were told to keep to the exact wording of the questionnaire, not help the women chose the answer, and to keep the same tone of voice when reading all possible responses. Before both interviews, the women were told that there is no correct or incorrect answer, that all answers are acceptable, and that their answers would be kept completely anonymous.

For the first interview, the response rate was 90%—92.6% for Arab women and 87.6% for Jewish women. Altogether 501 women were approached and 450 were interviewed (2**2**5 Jewish women and 2**25** Arab women). When approached 3 months later, 9.97% (23 Arab women and 22 Jewish women) of those interviewed did not answer the follow-up survey. Therefore, the response rate for the second interview was also 90%. This left 203 Arab women and 202 Jewish women in the follow-up sample. The non-respondent women were not significantly different from those that agreed to answer the questionnaire 4 months after childbirth regarding age, education, income, work status, number of children and obstetric characteristics.

The questionnaire included 9 parts: socioeconomic and demographic status, obstetric characteristics, social networks and support, negative social interactions, perceptions of customs and traditions intended to help the mother cope after childbirth, marital satisfaction, sense of parental competence, breastfeeding and health outcomes. The questionnaire was developed based on previous questionnaires and qualitative interviews with mothers from these population groups [22]. The original Hebrew questionnaire was translated into Arabic and back-translated into Hebrew to assure the correct translation. A pretest was performed for both the Hebrew and Arabic versions of the questionnaire. Very small changes were made to the two versions as a result of the pretest. The second interview utilized a shorter version of the questionnaire with a focus on health outcomes.

### Variables measured

#### Socioeconomic and demographic status

Included age, education, ethnic group (Arab or Jewish), years of marriage, work status (work out of home or not), income (above average, average or below average—compared to Israel’s net mean household income of 14,000 New Israeli Shekels), and place of residence (town or village).

#### Obstetric characteristics

Described if the pregnancy was achieved naturally or with medical help, if the delivery was normal or assisted (vacuum, forceps, elective or emergency caesarean section), if the mother fully or partially breastfed 1 month after childbirth, and number of children.

#### Social support

A social support scale was created by combining 11 items from Sherbourne & Stewart (1991) that were adapted to adequately measure social support after childbirth, and four items that were developed using findings from the initial qualitative study. The scale included three items depicting emotional support and eight items depicting instrumental support (See supplementary file 1).

The four additional items measured instrumental social support and inquired about having someone to help the mother take care of her older children, help take care of the baby, help so the mother could sleep and teach the mother how to take care of the baby and herself.

The available answers were on a range of five, from ‘never’ (1) to ‘always’ (5). Cronbach’s alpha was 0.84, 0.82 and 0.89 for instrumental, emotional and general social support (mean of both types of support) among Jewish women respectively, and 0.92, 0.85, and 0.92, respectively among Arab women.

#### Negative interactions

The 19-item negative interaction questionnaire was based on Brooks and Dunkel’s 2011 model of social negativity [15]. The questionnaire used a five-point Likert scale and included three dimensions: conflicts (8 items), insensitivity (7 items), and interference (4 items). The items were based on preliminary qualitative interviews and two previous studies on social support [24, 25, 22]. Principal component factor analysis was performed. Three components emerged fitting the theoretical model. Cronbach’s alpha was calculated separately for each component and for the entire scale. Among Jewish women, Cronbach’s alpha values were 0.95, 0.94, 0.83, and 0.96 for conflicts, insensitivity, interference, and the total scale, respectively. For Arab women, they were 0.94, 0.94, 0.74 and 0.96 respectively. Principal component analysis was performed with Varimax rotation. The analysis fitted the theoretical sub groups in both languages (Hebrew and Arabic) explaining 67.4 and 64% of variance respectively. (See supplementary file 1).

#### Sources of support and interactions

The women were asked to rate the levels of support and negative interactions they experienced from the various people around them—spouse, family, the spouses’ family, friends, neighbors and acquaintances—on a five-level scale from ‘does not help at all’ (1) to ‘is very helpful’ (5). (See supplementary file 1).

#### Perceptions of customs

This variable described how the women were impacted by postpartum customs and was measured using 19 items developed from the qualitative interviews. Women had to rate their agreement with the items (i.e. “To what extent do the customs and traditions that are performed for postpartum women enable you to rest” and “…cause you to be stressed”) on a scale of five from ‘very much’ (1) to ‘not at all’ (5). Four items were in the opposite direction and were reversed for analysis. Cronbach’s alpha was 0.84 among Jewish women and 0.90 among Arab women. (See supplementary file 1).

#### Self-reported health (SRH)

The women were asked to rate their current general health on a four-level scale, from very good to not good. For the logistic regression analysis, very good health was coded as 1 and the other three levels were coded as 0, as a high percentage of women (76% of Jewish women and 67% of Arab women) rated their health as very good and only a small percent as less than very good.

#### Health problems

The women were presented with a list of 12 problems and asked to rate if they suffered from each problem, on a five-level scale, from ‘not at all’ (1) to ‘very much’ (5). The problems included: muscle pain, stomach pain, lower back pain, neck and shoulder pain, problems sleeping, pain in genitalia, constipation, emotional exhaustion, and feeling worried, cross and stressed [1]. A mean score was calculated 4 months after childbirth with a Cronbach’s alpha of 0.91 for Jewish women and 0.83 for Arab women. (See supplementary file 1).

### Statistical analysis

Analyses were performed with SPSS V21. T-test, chi^2^ tests, Man-Whitney and Wilcoxon tests were used to measure differences between Arab and Jewish women and between the first month and fourth month after childbirth. Spearman’s correlations were used to examine the relationship between health outcomes and social support, negative interactions and perceptions of customs and traditions. Multivariate linear regression models were run to assess the variables predicting health problems 4 months after childbirth and logistic regression models were run to assess factors predicting SRH 4 months after childbirth.

## Results

Jewish (*n* = 202) and Arab (*n* = 203) women were interviewed 1 month (four to 6 weeks) after childbirth and 4 months after childbirth. Table 1 presents socio-demographic characteristics, obstetric characteristics and statistical differences between the two ethnic groups. The Arab women were about 2 years younger than the Jewish women. All were married with a mean duration of 5 years and there was no difference in number of children between the two groups. A higher percentage of Jewish women worked out of the home and had an academic education whereas, a higher percentage of Arab women breastfeed a month after childbirth. All the women reported being healthy and having a healthy baby born after the 37th week of pregnancy.

**Table 1:** Socioeconomic and obstetric characteristics of the study population by group (Mean, Standard Deviation or Percent)

Variable		Jews (*N* = 202)	Arabs (*N* = 203)			χ^2^	*p*
		M	SD	M	SD		
	Age	29.5	4.25	27.5	4.81		< 0.001
	Years of marriage	4.83	3.81	5.45	4.22		0.12
Socioeconomic Characteristics							
		N	%	N	%		
Education	High School	76	37.4	93	46.0	0.12	0.057
	Professional education	33	16.2	39	19.3		
	Academic education	94	46.3	70	34.6		
Work outside the home	Yes	133	65.5	74	36.6	0.29	< 0.001
	No	69	34	127	62.9		
Income	Above average	46	22.7	36	17.8	0.13	0.04
	Equal to average	53	26.1	39	19.3		
	Below average	99	48.8	125	61.9		
Place of residence	Town	150	73.9	139	68.8	0.09	0.26
	Village	53	26.1	63	31.2		
Obstetric Characteristics							
Pregnancy	Spontaneous	190	93.6	192	95.0	0.03	0.53
	Fertility treatment	13	6.4	10	5.0		
Type of Delivery	Normal vaginal delivery	173	85.6	160	79.2	0.01	0.08
	Assisted delivery	29	14.4	42	20.8		
Breastfeeding one month after birth	Yes^a^	157	77.3	184	91.1	0.22	< 0.0001
	No	46	22.7	18	8.9		
Number of children	1	51	25.1	50	24.8		0.41
	2	81	39.9	67	33.7		
	3	44	21.7	45	21.8		
	4	16	7.9	28	13.9		
	5 and above	11	5.4	9	4.5		

Note. The statistical tests included T and chi^2^ tests to assess the difference between Jewish and Arab women

^a^Indicates full or partial breastfeeding one month after child birth

Social support and negative social interactions 1 month after childbirth are presented in Table 2. No difference in the level of emotional support was found between the two groups, however, Jewish women reported significantly higher levels of general positive social support and instrumental positive support. Both Arab and Jewish women received high levels of support from their families, however, Jewish women reported significantly higher levels of support from their partners than Arab women.

**Table 2:** Social support, negative interactions and perceptions of customs among Jewish and Arab women, one month after childbirth (Mean, Standard Deviation, Man-Whitney Test)

Variable		Jews (*N* = 202)		Arabs (*N* = 203)		*p*
		M^a^	SD	M^a^	SD	
Social support	General positive support	4.27	0.73	3.79	0.87	< 0.001
	Emotional support	4.53	0.64	4.46	0.72	0.67
	Instrumental support	4.15	0.72	3.53	0.92	< 0.001
Sources of social support	Partner	4.57	0.71	3.95	0.99	< 0.001
	Woman’s family	4.32	0.91	4.22	0.83	0.09
	Husband ‘s family	2.74	0.61	2.62	0.93	0.29
	Friends	2.66	0.69	2.54	0.92	0.14
	Neighbors	2.30	0.87	2.15	0.90	0.12
Negative interactions	Average	1.47	0.74	1.82	0.85	< 0.001
Subscale of negative interactions	Conflict	1.68	1.03	1.99	1.09	< 0.001
	Insensitivity	1.36	0.68	1.74	0.88	< 0.001
	interference	1.24	0.54	1.59	0.70	< 0.001
Source of negative interactions	Partner	1.43	1.14	1.49	1.04	0.047
	Woman’s family	1.35	0.96	1.38	0.87	0.105
	Husband ‘s family	1.92	1.31	2.34	1.33	< 0.001
	Friends	1.13	0.59	1.15	0.55	0.159
	Neighbors	1.12	0.57	1.14	0.52	0.167
Perception of customs and traditions		4.34	0.54	3.13	0.83	< 0.001

Note. Differences between Arab and Jewish women were measured using the Man-Whitney Test

^a^*Range 1–5*

When asked about the negative interactions, Arab women reported experiencing significantly higher levels of negative interactions for all three subcategories: conflict, insensitivity, and interference. Among the Arab women, the main source of these negative interactions was the husband’s family. Neighbors, friends, family, and partners did not serve as major sources of negative interactions for either group.

Considering the women’s perceptions of traditions and customs intended to offer support after childbirth, Jewish women had significantly more positive perceptions than Arab women (mean = 4.34 and mean = 3.13 respectively).

Table 3 presents the two self-report health outcomes measured at both one and 4 months after childbirth. Overall, Arab women reported worse health compared to Jewish women.

**Table 3:** Self-reported health outcomes one versus four months after childbirth in Jewish and Arab women (Mean, SD, T-Test, Wilcoxon Test)

Variable		Jews (*N* = 202)				*p*	Arabs (*N* = 203)				*p*
		1 Month		4 Months			1 Month		4 Months		
		M	SD	M	SD		M	SD	M	SD	
Health^a^ problems	Mean	2.44	0.84	2.27	0.77	0.001	2.59	0.44	2.36	0.63	< 0.001
	Physical	2.63	0.93	2.43	0.86	< 0.001	2.75	0.41	2.51	0.67	< 0.001
	Mental	2.04	0.90	1.93	0.80	< 0.001	2.26	0.70	2.07	0.80	< 0.001
Self-Reported Health^b^		3.77	0.45	3.74	0.48	0.28	3.53	0.54	3.62	0.57	0.005

Note. Changes in health between one and four months were measured within each group using paired t-tests for health problems and Wilcoxon’s tests for self-reported health

^a^ *Range 1–5* ^b^ *Range 1–4*

The first measure included mental and physical health problems. A mean score for 12 health problems was calculated on a range of 0–5. The number of health problems reported declined significantly from the first to the second interview among both groups. Arab women reported significantly higher levels of mental problems 1 month after childbirth compared to Jewish women (mean = 2.26 and mean = 2.04, respectively). There were no other significant differences in health problems between the two groups at either time point.

Between the first and fourth month after childbirth, self-reported health (SRH) improved significantly among Arab women but remained the same for Jewish women who had significantly higher SRH than Arab women at both time points.

Associations between social support, negative social interactions and perceptions of customs at 1 month after childbirth and health outcomes 4 months after childbirth are shown in Table 4. Among both Jewish and Arab women, social support was associated negatively with health problems and positively with SRH, suggesting that higher levels of social support predict better health. On the other hand, negative interactions had the opposite effect for both groups, suggesting that women that experience negative interactions at 1 month have worse health 4 months after childbirth.

**Table 4:** Associations between social interactions at one month postpartum and health measures at four months postpartum in Jewish and Arab women (Spearman correlations)

	Jews (*N* = 202)		Arabs (*N* = 203)	
	Health problems	Self-reported health	Health problems	Self-reported health
Social support	−0.39**	0.32**	−0.32**	0.39**
Negative interactions	0.32**	−0.33**	0.49**	−0.55**
Conflict	0.31**	−0.33**	0.48**	−0.55**
Insensitivity	0.30**	− 0.25**	0.50**	− 0.50**
Interference	0.32**	−0.25**	0.37**	−0.43*
Perception of customs and traditions	−0.16*	0.28*	−0.52*	0.38**

*p < 0.05 **p < 0.001

Perceptions of customs and traditions were associated with both health measures in both groups. Women that reported more positive perceptions of customs 1 month after childbirth also reported better SRH and lower levels of health problems 4 months after childbirth. These associations were stronger among Arab women.

Multivariate regressions, with the health measures as the dependent variables, were performed to control for other variables (Tables 5 & 6). Overall, both social support and negative interactions 1 month after childbirth predicted health outcomes at 4 months in opposing directions after adjusting for age, education, type of delivery and population group with notable differences being found between the two groups.

**Table 5:** Variables predicting self-reported health four months after childbirth (multivariate logistic regression)

Variable		Total population				Jews				Arabs		
	OR	95% CI		*p*	OR	95% CI		*p*	OR	95% CI		*p*
		LL	UL			LL	UL			LL	UL	
Social support	1.81	1.30	2.52	< 0.0001	2.33	1.38	3.93	0.002	1.59	1.01	2.49	0.04
Negative interactions	0.53	0.38	0.74	< 0.0001	0.79	0.48	1.31	0.37	0.32	0.19	0.54	< 0.0001
Perception of customs	1.98	1.33	2.97	0.001	2.85	1.39	5.82	0.004	1.48	0.88	2.51	0.14
Age	1.04	0.99	1.10	0.13	1.11	1.01	1.23	0.03	0.99	0.92	1.07	0.89
Education	1.39	1.03	1.86	0.03	1.84	1.19	2.83	0.006	1.03	0.66	1.59	0.90
Type of delivery	1.22	0.62	2.39	0.56	2.07	0.64	6.73	0.23	1.00	0.41	2.42	0.99
Population group	3.02	1.43	6.35	0.004								
		*x*^2^_(7)_ = 98.3; *p <.*000 *Nagel* ker *ke* - *R*^2^ = 0.31				*x*^2^_(6)_ = 44.37 *p <.*000 *Nagel* ker *ke* - *R*^2^ = 0.29				*x*^2^_(6)_ = 66.8; *p <.*000 *Nagel* ker *ke* - *R*^2^ = 0.38		

*LL* Lower limit; *UL* Upper limit

**Table 6:** Variables predicting health problems four months after childbirth, linear multivariate regression

Variable	Total population			Jews			Arabs		
	B	Beta	*p*	B	Beta	*p*	B	Beta	*p*
Social support	−0.20	− 0.23	< 0.0001	− 0.37	−0.34	< 0.0001	− 0.07	− 0.097	0.145
Negative interactions	0.24	0.28	< 0.0001	0.21	0.20	0.003	0.28	0.37	< 0.0001
Perception of customs	−0.17	−0.22	0.001	−0.18	−0.13	0.045	−0.19	− 0.25	< 0.0001
Age	−0.002	− 0.02	0.74	− 0.01	− 0.07	0.28	0.008	0.06	0.28
Education	0.09	0.12	0.01	0.06	0.07	0.25	0.11	0.15	0.01
Type of Delivery	0.29	0.15	< 0.0001	0.46	0.21	0.001	0.13	0.09	0.15
Population group	−0.29	−.207	< 0.0001						
	*F*(8,577) = 23.80; *p <.*001 *R*^2^ = 0.29			*F*(6,247) = 14.56; *p <.*001 *R*^2^ = 0.31			*F*(5,003) = 19,27; *p <.*001 *R*^2^ = 0.37		

Social support significantly predicted better SRH in both Jewish and Arab women (OR 2.33, CI 1.38–3.93 and 1.59, CI 1.01–2.46; Table 5), however, social support only predicted fewer health problems among Jewish women (Table 6). An opposite picture was observed for negative social interactions. Negative interactions predicted higher levels of health problems in both Arab and Jewish women (Table 6). As well, higher levels of negative interactions predicted lower levels of SRH among Arab women (OR 0.32, CI 0.19–0.54; Table 5). However, this association was not observed among Jewish women. Positive perceptions of customs and traditions were associated with higher SRH in Jewish women and fewer health problems in both groups (Tables 5 & 6).

In a regression model including both population groups, Jewish women have significantly lower levels of health problems and a higher chance of reporting better SRH compared to Arab women, after adjusting for all other variables.

## Discussion

The present study explored the relationship between social support, negative social interactions and perception of customs and maternal health in Arab and Jewish women. By establishing temporality, this study was able to build upon the literature by providing evidence for a predictive relationship between social support, negative social interactions and perception of customs and both mental and physical health.

In general, postpartum women who experienced higher levels of social support reported fewer health problems and better SRH, with the opposite being true for negative social interactions.

Among Jewish women, social support was found to predict better SRH and fewer health problems. However, among Arab women social support only significantly predicted SRH, not health problems, suggesting that social support may be more important for predicting health problems among Jewish women.

The literature extensively reinforces the finding that social support positively impacts women’s physical and mental health in the postpartum period [3, 26, 27, 28]. However, it is important to note that individualist societies have had greater representation in the literature, which may explain the lack of reflection of these associations among the Arab women in the present study.

The finding that negative interactions predict higher levels of health problems among both Arab and Jewish women was supported by the literature [29]. Studies have shown negative interactions to be associated with physiological responses that increase the risk of developing poor health, decreased feelings of self-control and self-esteem, and increased mood and anxiety disorders [30, 31].

For Arab women, it was also found that negative interactions predict lower levels of SRH. This association was not present in Jewish women, indicating that negative interactions may be more influential on health among Arab women. Interestingly, the negative impact of negative interactions on health was found to be greater than the positive impact of social support among Arab women. Studies have found that although negative interactions usually occur less frequently than positive support, they may significantly reduce health and well-being and have a greater impact on mental health than social support [32, 33, 34].

Perception of customs had an inverse relationship with health problems, with more positive perceptions predicting fewer health problems and vice versa in both groups. The associations were stronger in Arab women, which may be a result of the magnitude and intensity of traditions practiced, as described in the introduction.

As noted, Arab society in Israel is going through a transition from traditional to more modern values. A recent study by Emad Gith (2018) [12], looked at postpartum depression (PPD) in traditional and modern Arab Israeli women. Almost all of the women with PPD were found to be closer to the modern end of the spectrum with the severity of PPD increasing with modernism. These findings are explained by the interpersonal conflict that accompanies dissonance between the views of new mothers and older generations, particularly with the husband’s mother and family. It is important to note that the present study considered mental health outside of the context of PPD, highlighting the presence and importance of other mental health symptoms and their linkage to social interactions and cultural customs.

Preliminary qualitative research for this study revealed that many Arab women perceived their husbands’ families as very powerful and controlling and felt it difficult to go against their wishes, resulting in conflict with the mother-in-law and with their husbands [22]. Other studies have also found the relationship with the mother-in-law to have a large impact on the health of new mothers, revealing mental and physical health, marital conflict, and maternal self-efficacy to be associated with mother/daughter-in-law conflicts [19, 11, 35, 36].

By large, the Jewish society in Israel is modern and individualistic. Thus, individuals are seen as independent and autonomous with personal needs being valued before those of the group [12, 37]. This, along with more “traditional notions of family life” [38] (p.574) among Arab society, may be linked to the findings of the present study, in that Jewish women have more authority over how they interact with others and the customs that they participate in [39]. Their ability to dictate the terms in which their social interactions occur is likely linked to their more positive perceptions and the lesser extent to which they are affected by interactions and customs. For example, in the preliminary research Jewish women reported that they would freely tell their mother in law if they did not want her to visit whereas, Arab women reported that they felt unable to do so.

In addition to these primary findings, important differences were also found between Arab and Jewish women in demographics and secondary outcomes. Arab women were on average younger and less likely to work outside of the home or have an academic level education than Jewish women, reinforcing differences in roles and expectations of women in these two cultures [12]. Arab women had significantly lower levels of SRH and more health problems at 1 month and 4 months after giving birth when compared to Jewish women. Arab women’s lesser experience of social support—shown to protect emotional, physical and mental health after childbirth [26, 27, 28] may explain the poorer health outcomes among Arab women.

Sharing of anxieties and social support are protective of physical and mental health problems [40, 41]. It is possible that Arab women are less able to share their feelings openly due to cultural norms. In the Arab society, individual successes and failures reflect strongly on the family unit which may result in the suppression of emotions and constraint of personal desires [12, 37].

Additionally, it is possible that the support the Arab women received was not always in line with their needs. Previous research has shown that the positive influence of social support is negated if the support received is not reflective of the women’s true needs [42]. Although in transition to a more modern society, Arab society is often still traditional in family structure and the female role. Women are below their husbands in the family hierarchy and are expected to support and cater to his needs and the needs of his family [43, 39, 44]. Thus, if they are in objection to the support and cultural traditions being imposed on them by their husband’s family, they are often not empowered to say so.

### Strengths, limitations and future directions

The prospective nature of the study established temporality allowing for the predictive nature of the independent variables to be identified. Other study strengths include the matching of groups for SES, marital status, and absence of PPD and the use of multivariate analysis to control for potential confounders.

As researcher bias can influence research study directions and conclusions [45], actions were taken to minimize the effect, such as training research assistants to use consistent wording and tone and allow respondents to come to conclusions on their own. Additionally, the data was analysed and interpreted by researchers from three different ethnocultural and professional backgrounds, limiting the potential for researcher bias to influence the study’s conclusions.

It is important to note that the findings of this study are limited, in that, they may not be representative of all Jewish and Arab women in or outside of Northern Israel and important differences may exist at the individual or community level. Further research is warranted to understand the extent to which these findings apply in other areas of Israel and abroad.

In addition, future research should look to expand upon this knowledge and work to identify ways in which to decrease the negative interactions that exist in women’s lives after giving birth. Potential strategies include facilitating the empowerment of Arab women, especially in the context of their health, and finding ways to increase opportunities and channels for social support for these women.

## Conclusions

In conclusion, these findings highlight the impact of both negative and positive social interactions and cultural and societal context on postpartum maternal health. Health care practitioners should be aware of this relationship and should work to be understanding of, and sensitive to, the cultural differences and social circumstances of their patients in order to ensure they receive the highest level of care. It is important for researchers and practitioners to continue to look deeper into the cultural context from which patients accessing public health care systems arise in order to provide effective and equitable care.

## Supplementary information

**Additional file 1.**

## Data Availability

The datasets generated during and/or analysed during the current study are available from the corresponding author on reasonable request. They are in Hebrew.

## Abbreviations

SRH: Self-reported health
OR: Odds ratio
CI: Confidence interval
MOH: Ministry of health
MCHC: Mother and child health clinics
SES: Socio-economic status
PPD: Postpartum depression
