# Supplementary material for: Health may be compromised by social interactions depending on culture among postpartum Arab and Jewish Israeli women
Source: BMC Pregnancy Childbirth. 2020 Aug 21;20:480. doi: 10.1186/s12884-020-03168-4 (PMC7441553; doi:10.1186/s12884-020-03168-4)
Supplement: Supplementary file 1 — Additional file 1. [file 12884_2020_3168_MOESM1_ESM.docx]

**Supplementary file 1**

**Translation of the Hebrew questionnaire**

*Social support*

At what rate have these situations occurred during the last month, please rate on a 1 to 5 scale where 5 represents “very frequent” and 1 “not at all”:

1. There is someone to listen to me when I need to talk about my feelings
2. There is someone to give me love and affection
3. There is always someone around when I need help
4. There is someone to make me a meal if I did not have time
5. There is someone to help me take care of my older children (if there are older children)
6. There is someone to help take care of the baby
7. There is someone to help and let me sleep
8. There is someone to help me with house work
9. There is someone to help do things I need to do
10. There is someone to teach me how to take care of the baby and myself
11. There is someone to help me financially

*Negative interactions.*

During the last month, rate the frequency these situations describe a person or people that try to help you, please rate on a 1 to 5 scale where 5 represents “very frequent” and 1 “not at all”:

1. I argued with this person
2. I had to work hard not to confront this person
3. I want very much this person to change
4. This person does not make me feel good
5. This person hurts my feelings
6. Problems in this relationship are not solved
7. He/she were not sensitive to my needs
8. He/she did not take my whishes into consideration
9. He/she are rude to me
10. He/she are critical of what I do
11. He/she caused me to be stressed
12. He/she disregarded my opinions and did not listen to them
13. He/she gave unwanted or wrong advice
14. He/she intruded on my family territory
15. He/she intruded into privet aspects of my life
16. He/she go on giving help when it is not needed
17. He/she do not let me be independent with activities with my baby
18. He/she tried to convince me to do things I do not want to do
19. He/she tries to control or influence my life

*Sources of support and interactions*.

Please rate the extent you got help during the last few months from the following people:

Spouse, family, the spouses' family, friends, neighbors and acquaintances—on a five-level scale from ‘not relevant-0, not help at all’ (1) to ‘very much’ (5).

Please rate the extent this person (or people) were a source of these feelings:

Spouse, family, the spouses' family, friends, neighbors and acquaintances—on a five-level scale from ‘not relevant -0, not help at all’ (1) to ‘very much’ (5).

*Perceptions of customs*.

In many communities there are customs and traditions that are performed to help women after childbirth. Please rate your agreement with the following items on a scale of five, from ‘very much’ (5) to ‘not at all’ (1):

1. The customs and traditions enable me to rest
2. They are a source of mental support at this important time
3. They make my physical pain worse
4. They make me stressed
5. People come for visits without an invitation
6. I need to perform extra tasks such as preparing the house and hosting
7. The customs present an extra financial burden at this time
8. If I did not have to perform the customs and traditions I would have given them up
9. They decrease my efficiency with the house work
10. They disturb me from looking after my baby
11. They disturb me breastfeeding
12. They disturb me from looking after my older children
13. They have a bad influence on my relationship with my older children
14. They hurt my relationship with my husband
15. They do not let me rest
16. They let me get more sleep
17. They disrupt my privacy
18. They help me do the chores I need to do
19. I could get along without these customs and traditions

*Health problems*.

To what extent did you suffer from these problems during the last two months: on a five-level scale, from ‘not at all’ (1) to ‘very much’ (5).

The problems included:

Muscle pain, stomach pain, headaches, lower back pain, neck and shoulder pain, problems sleeping, pain in genitalia, constipation, emotional exhaustion, feeling worried, cross and stressed.
